# Supplementary material for: Denouncing the use of field-specific effect size distributions to inform magnitude
Source: PeerJ. 2021 Jun 14;9:e11383. doi: 10.7717/peerj.11383 (PMC8210805; doi:10.7717/peerj.11383)
Supplement: Supplemental Information 3 — We open a fake dataset that contains different values of Cohen’s d and Pearson’s r: the standardized mean difference and the correlation coefficient, respectively. This data has characteristics that are similar to Hemphill (2003) but they could just as easily be studies in another sub-discipline or an entire field (e.g., Psychology). [file peerj-09-11383-s003.pdf]

Denouncing the Use of Field-Specific Effect Size Distributions to Inform Magnitude

Empirical Distribution of Effect Sizes - Demonstration

This is an example of how effect size distributions are used to interpret magnitude. We include this example for readers to be able to recognize this method and avoid it.

We open a fake dataset that contains different values of Cohen's d and Pearson's r: the standardized mean difference and the correlation coefficient, respectively. This data has characteristics that are similar to Hemphill (2003) but they could just as easily be studies in another sub-discipline or an entire field (e.g., Psychology).

```
library(ggplot2)
```

```
## Warning: package 'ggplot2' was built under R version 4.0.2
```

```
fakedat <- read.csv("fakedat")
names(fakedat)
```

```
## [1] "X" "d" "r"
```

```
# plot (Cohen's d)
ggplot(fakedat, aes(x = d)) +
  geom_histogram(binwidth= .25, colour="black", fill="blue", alpha = .5) +
  labs(title = "Effect Size Distribution") +
  ylab("Frequency") + xlab("Effect Size (Cohen's d)") +
  theme_minimal()
```

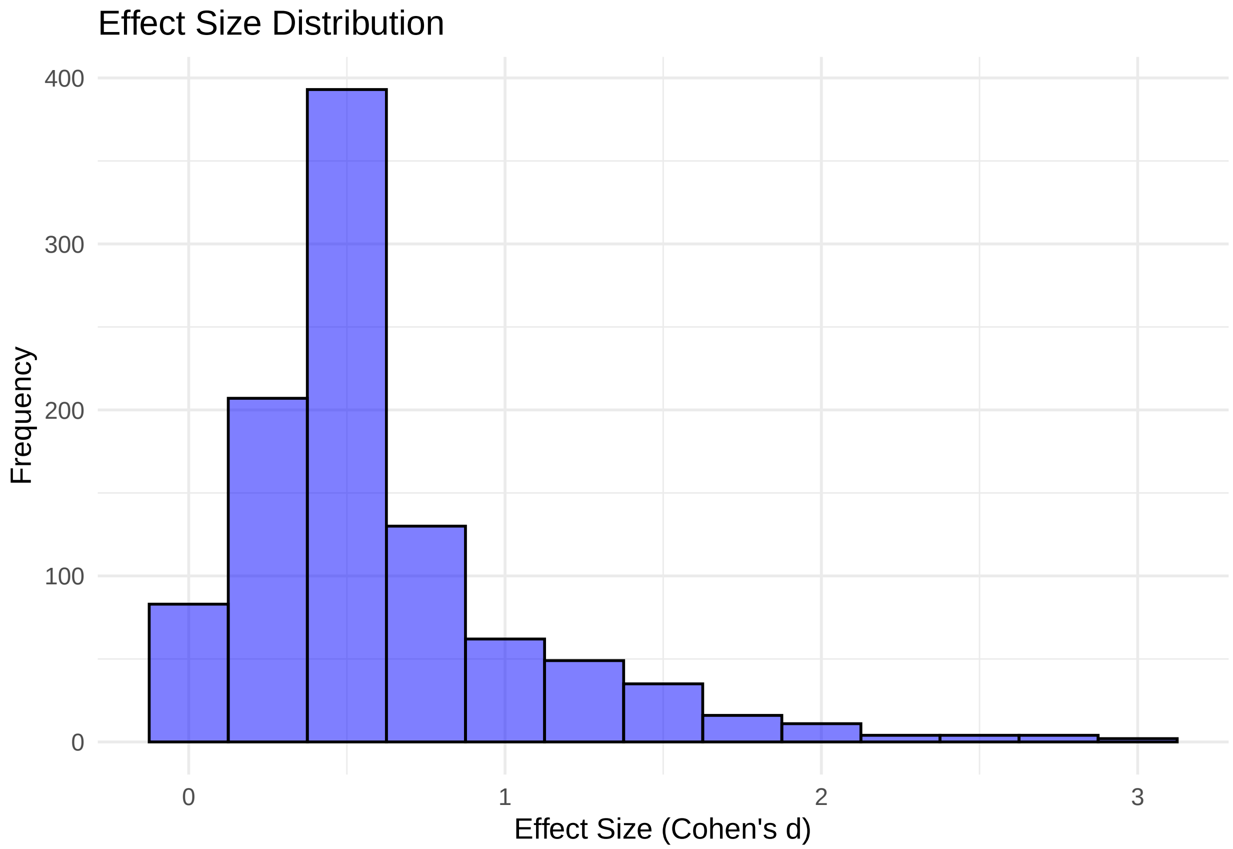


```r
# plot (Pearson's r)
ggplot(fakedat, aes(x = r)) +
  geom_histogram(binwidth= .25, colour="black", fill="orange", alpha = .5) +
  labs(title = "Effect Size Distribution") +
  ylab("Frequency") + xlab("Effect Size (Pearson's r") +
  theme_minimal()
```

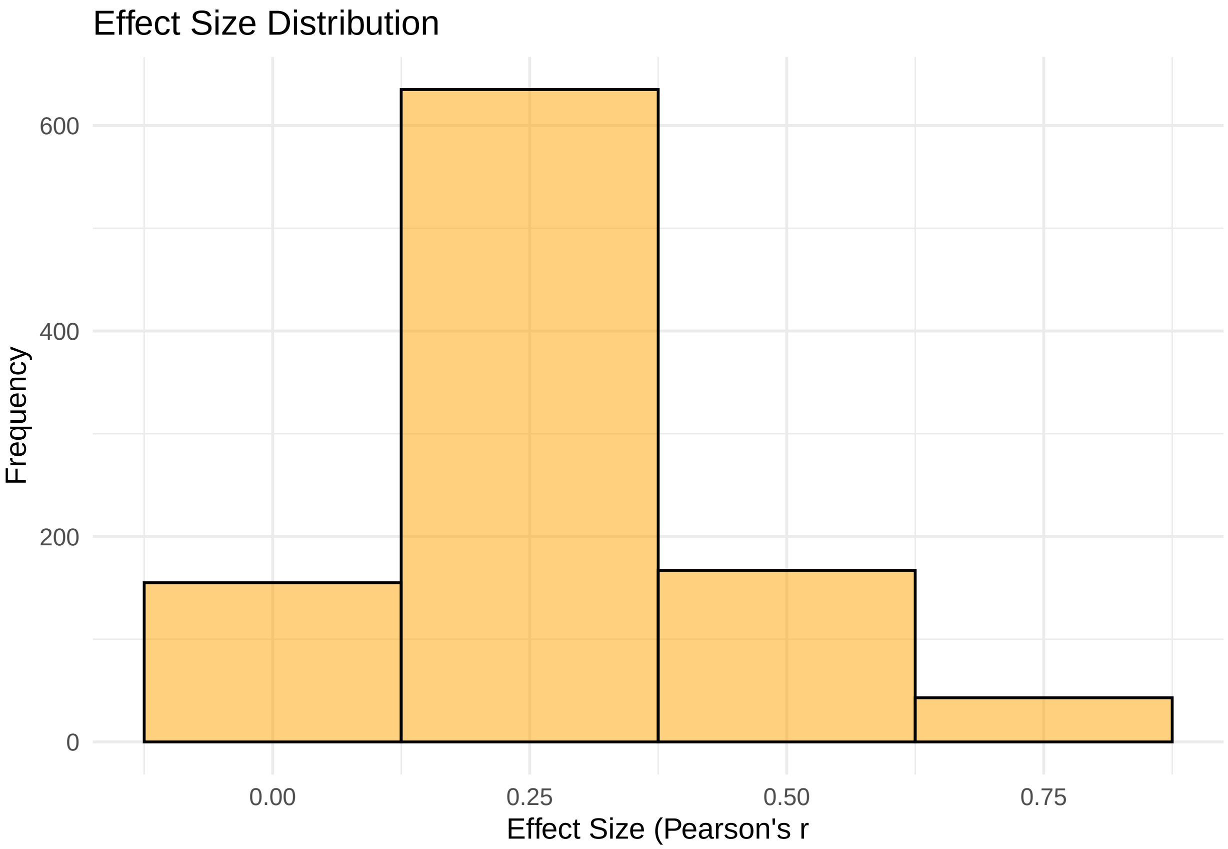

Next, we obtain the small, medium, and large label effect sizes by splitting each distribution into thirds.

```
round(quantile(fakedat$d, prob = c(0.33, 0.66)), 2)
```

```
## 33% 66%
## 0.4 0.6
```

```
round(quantile(fakedat$r, prob = c(0.33, 0.66)), 1)
```

```
## 33% 66%
## 0.2 0.3
```

The cut-offs in this fake dataset match those of Hemphill (2003). For Cohen's *d*, a small effect is an effect that is smaller than 0.40, a medium effect is between 0.40 and 0.60, and a large effect is greater than 0.60. For Pearson's *r*, a small effect is an effect that is smaller than 0.2, a medium effect is between 0.2 and 0.3, and a large effect is greater than 0.3. Thus, a researcher would compare their own effect size (standardized mean difference or correlation) to these cut-offs to determine if their effect size is "small", "medium", or "large".

Once again, this demonstration is only meant to help researchers detect this method. We discourage its use for reasons outlined in the manuscript.
